# Impact of contractile reserve on acute response to cardiac resynchronization therapy

**DOI:** 10.1186/1476-7120-6-65

**Published:** 2008-12-31

**Authors:** Marie Moonen, Mario Senechal, Bernard Cosyns, Pierre Melon, Eric Nellessen, Luc Pierard, Patrizio Lancellotti

**Affiliations:** 1 Department of Cardiology, CHU Sart Tilman, Liege, Belgium; 2 2725, chemin Sainte-Foy, Québec, Canada; 3 Chirec Hôpital de Braine-l'Alleud Waterloo, rue Wayez 35, 1420 Braine l'Alleud, Belgium

## Abstract

**Background:**

Cardiac resynchronization therapy (CRT) provides benefit for congestive heart failure, but still 30% of patients failed to respond to such therapy. This lack of response may be due to the presence of significant amount of scar or fibrotic tissue at myocardial level. This study sought to investigate the potential impact of myocardial contractile reserve as assessed during exercise echocardiography on acute response following CRT implantation.

**Methods:**

Fifty-one consecutive patients with heart failure (LV ejection fraction 27% ± 5%, 67% ischemic cardiomyopathy) underwent exercise Doppler echocardiography before CRT implantation to assess global contractile reserve (improvement in LV ejection fraction) and local contractile reserve in the region of the LV pacing lead (assessed by radial strain using speckle tracking analysis). Responders were defined by an increase in stroke volume ≥ 15% after CRT.

**Results:**

Compared with nonresponders, responders (25 patients) showed a greater exercise-induced increase in LV ejection fraction, a higher degree of mitral regurgitation and a significant extent of LV dyssynchrony. The presence of contractile reserve was directly related to the acute increase in stroke volume (r = 0.48, p < 0.001). Baseline myocardial deformation as well as contractile reserve in the LV pacing lead region was greater in responders during exercise than in nonresponders (p < 0.0001).

**Conclusion:**

The present study showed that response to CRT largely depends not only on the extent of LV dyssynchrony and the severity of mitral regurgitation but also on the presence of contractile reserve.

## Background

The number of patients presenting with heart failure is increasing rapidly. Currently, cardiac resynchronization therapy (CRT) has emerged as an adjunctive treatment in patients with drug-refractory heart failure [1]. The clinical benefit of CRT – evidenced by improvement in symptoms, quality of life, exercise capacity, and left ventricular (LV) systolic performance – has been largely demonstrated [2,3]. Acutely, a CRT-increase in forward stroke volume by ≥ 15% has been shown to predict long-term clinical outcome in both ischemic and non ischemic cardiomyopathy [4]. However, 20% to 30% of patients do not respond to CRT despite application of recommended selection criteria [5]. Therefore, additional selection criteria are needed. Response to CRT largely depends on the extent of LV dyssynchrony, the severity of of mitral regurgitation and the possibility offers to the LV to recruit function [6-9]. CRT response could thus correlate with myocardial viability and inversely be associated with the extent of myocardial fibrosis [10,11]. Furthermore, scar tissue or fibrosis – characterized by lack of contractile reserve – in the LV pacing lead region may prohibit response to CRT. Myocardial contractile reserve – important prognostic marker in heart failure – can be reliably assessed during exercise echocardiography [12]. This study sought to investigate the potential impact of myocardial contractile reserve as assessed during exercise echocardiography on acute response following CRT implantation.

## Methods

### Patient population

Consecutive patients (n = 51) with severe heart failure, scheduled for implantation of a permanent biventricular pacemaker, were included using established selection criteria for CRT: New York Heart Association class III or IV despite optimal medical therapy, LV ejection fraction ≤ 35%, QRS with left bundle branch block configuration or a duration >120 ms. The aetiologies of heart failure were idiopathic dilated cardiomyopathy in 17 patients and ischaemic heart disease in 34. All patients underwent exercise Doppler echocardiography before CRT implantation to assess global LV contractile reserve and local contractile reserve in the region of the LV pacing lead (assessed by longitudinal strain using speckle tracking analysis). No patients with ischaemic disease presented inducible ischaemia during test. The protocol was approved by the Human Ethical Committee of our University Hospital and all patients gave informed consent.

### Exercise echocardiography

A symptom-limited graded bicycle exercise test was performed in a semi-supine position on a tilting exercise table. After an initial workload of 25 W maintained for 2 min, the workload was increased every 2 min by 25 W. Blood pressure and a 12-lead electrocardiogram were recorded every 2 min. Two-dimensional echocardiographic recordings were made throughout the test.

### Echocardiographic measurements

All echocardiographic parameters were obtained at rest and at peak exercise in the same cycling semi-supine position (Vivid 7 imaging device, GE Healthcare, United Kingdom) and were obtained in digital format and stored on optical disks for off-line analysis. Two-dimensional grayscale images (frame rates ≥ 70 s^-1^) and colour-tissue Doppler imaging (frame rates ≥ 115 s^-1^) were performed in the apical views using a narrow sector angle. Left ventricular end-diastolic and end-systolic volumes and ejection fraction were measured by the biapical Simpson disk method. Wall motion score analysis was applied to a 16-segment model of the LV with a semiquantitative scoring system (1 = normal, 2 = hypokinesia, 3 = akinesia, 4 = dyskinesia). Quantitation of MR was performed by the proximal isovelocity surface area method as previously described [4]. Intra-LV dyssynchrony was referred to LV dispersion and was calculated as the difference between the longest and the shortest times to peak systolic velocities of 4 opposing basal walls (apical 2-and 4-chamber) [13]. Global LV contractile reserve was expressed as the change in LV ejection fraction from rest to peak exercise. Regional contractile reserve in the pacing lead was assessed using speckle tracking analysis from LV long axis images (mid segments). After tracing the endocardial borders in the end-systolic frame, an automated tracking algorithm outlined the myocardial deformation in the dedicated LV segments [14]. Peak systolic radial strain was only measured in the lateral, posterior and anterior regions, where the LV lead was positioned.

### CRT implantation and setting

All patients received a biventricular pacing device for CRT with a right ventricular apical lead and LV pacing electrodes positioned through the coronary sinus in a LV epicardial vein. This coronary sinus lead was placed in a lateral position in 32 patients, in a postero-lateral position in 16 and in anterior position in 3. After a successful implant, echocardiography was used to optimize the atrio-ventricular delay in order to maximize LV filling time. Interventricular pacing interval was set to default value (V-V = 0 ms).

### CRT response definition

Within the week after implantation, changes in forward stroke volume were assessed. Improvement was defined as an increase in stroke volume ≥ 15% after CRT [2] (see videos).

### Statistical analysis

Data are expressed as mean ± 1 SD (STATISTICA version 6). Student's paired 2-tailed t test was used to compare measurements obtained at rest and during exercise. Categorical variables were compared with Fisher exact test. A value of p < 0.05 was considered significant. Categorical data are summarized as frequencies and percentages. Linear regression analysis was performed to evaluate the relation between the improvement in LV ejection fraction during exercise and the percentages of changes in forward stroke volume after CRT implantation. The optimal improvement in LV ejection fraction and in myocardial strain during exercise to predict response to CRT was determined by receiver operating characteristic curve analysis. To detect independent cofactors associated with response to CRT, a logistic multivariate analysis was performed. All significant variables were included in the multivariate model. Reproducibility of echocardiographic measurements has been previously published [15].

## Results

### Responders versus non responders

**Figure 1:**
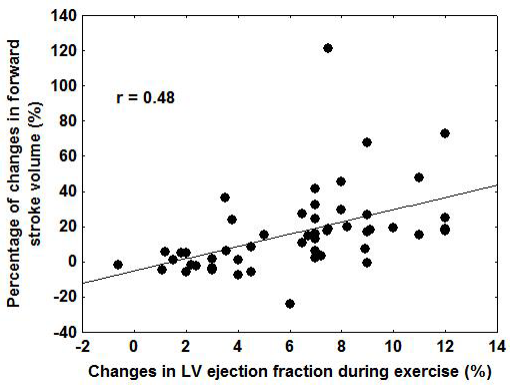
**Relationship between contractile reserve (improvement in LV ejection fraction during exercise) at inclusion and the percentage of changes forward stroke volume under CRT**.

**Figure 2:**
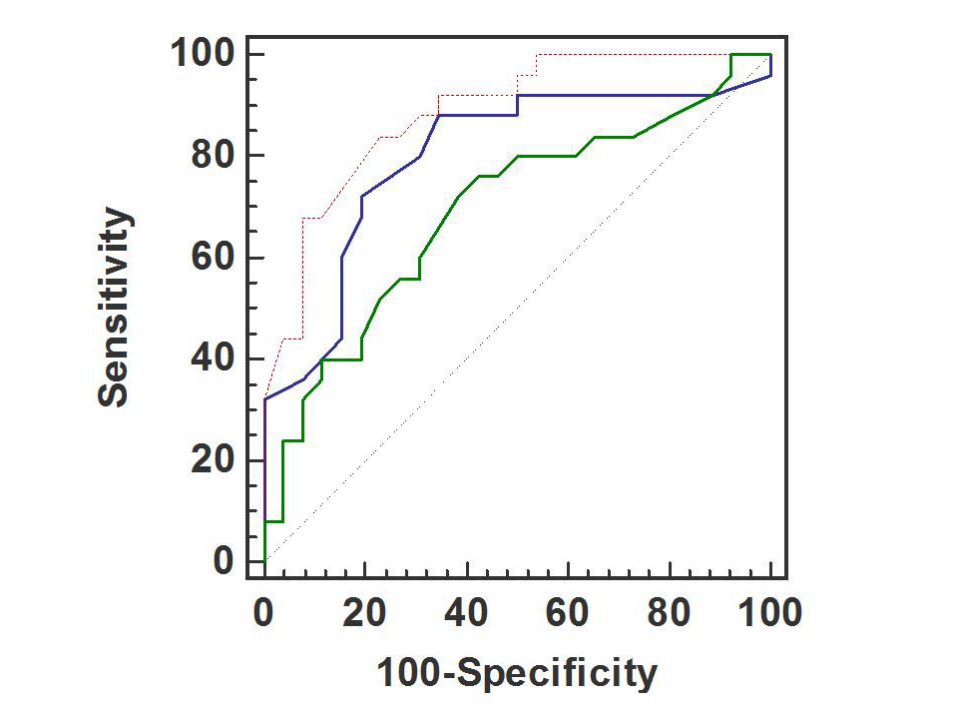
**Receiver operating characteristic curves analysis on various parameters to predict response after CRT**. Red: Changes in EF (ejection fraction), Blue: LV dyssynchrony, Green: mitral effective regurgitant orifice (ERO).

Within the 2 days after CRT implantation, 25 patients (49%) were responders. Baseline clinical and echocardiographic parameters between responders and non responders were similar, except for higher degree of mitral regurgitation, greater extent of LV dyssynchrony and better myocardial deformation by 2D speckle tracking in the LV lead region in responders (Table 1). During exercise, responders showed a greater increase in LV ejection fraction. Improvement in LV ejection fraction at peak test was correlated to the improvement in forward stroke volume after CRT (r = 0.48) (Figure 1). By multivariate logistic regression analysis, LV dyssynchrony (p = 0.017), the degree of mitral regurgitation (p = 0.028) and the presence of contractile reserve at exercise (p = 0.006) emerged as independent predictors of response to CRT. Table 2 depicted the area under the curves (ROC-curves) for these 3 independent predictors (Figure 2) (see additional files 1, 2, 3, 4 and 5).

**Table 1:** Responders versus non responders

**Variables**	**Responders** **(n = 25, 49%)**	**Non Responders** **(n = 26, 51%)**	**p**
Age, years	71 ± 8	69 ± 9	NS
Male, n (%)	15 (60)	17 (65)	NS
Ischemic cardiomyopathy, n (%)	15 (60)	19 (73)	NS
QRS duration, ms	154 ± 24	169 ± 25	NS
Diuretic, n (%)	19 (76)	23 (88)	NS
β-Blockers, n (%)	22 (88)	22 (85)	NS
ACEi, n (%)	19 (76)	22 (85)	NS
AR Blockers, n (%)	3 (12)	2 (8)	NS
Spironolactone, n (%)	9 (36)	14 (54)	NS
LV-RV dyssynchrony, ms	54 ± 16	50 ± 17	NS
LV dispersion, ms	109 ± 44	61 ± 35	<0.0001
Mitral effective regurgitant orifice, mm^2^	22 ± 11	15 ± 9	0.018
LV end-diastolic volume, ml	175 ± 34	193 ± 42	NS
LV end-systolic volume, ml	128 ± 26	139 ± 35	NS
LV ejection fraction, %	27.1 ± 5.0	27.7 ± 5.3	NS
LV ejection fraction at exer., %	35 ± 5.7	31 ± 5.3	0.03
LV ejection fraction diff., %	8.4 ± 2.4	4.2 ± 2.6	<0.0001
Wall motion score index	2.2 ± 0.27	2.28 ± 0.35	NS
Wall motion score index at exer	1.86 ± 0.34	2.15 ± 0.34	0.0033
Wall motion score index diff	-0.3 ± 0.26	-0.13 ± 0.21	0.013
Strain target LV lead wall, %	15 ± 2.8	10.2 ± 6.3	0.0007
Strain target LV lead wall at exer, %	19.3 ± 2.4	10.7 ± 8.5	<0.0001
Strain target LV lead wall diff., %	4.2 ± 1.6	0.44 ± 3.5	<0.0001

Ischemic cardiomyopathy, ACEi: angiotensin converting enzyme inhibitors and AR: angiotensin receptors, LV: left ventricle, RV: right ventricle. Diff: difference exercise-rest.

**Table 2:** Area under the curves, sensitivity, specificity and optimal cutoff values of predictors for acute CRT response.

**Data at inclusion**	**Cutoff values**	**AUC**	**Sensitivity**	**Specificity**
LV dyssynchrony	> 65 ms	0.80	88%	65.4%
Effective regurgitant orifice	> 15 mm^2^	0.69	76%	57.7%
Changes in LV ejection fraction at exercise	≥ 6.7%	0.89	84%	76.7%
Changes in wall motion score index at exercise	≥ 0.23	0.76	80%	69%

### Regional contractile reserve and response to CRT

**Figure 3:**
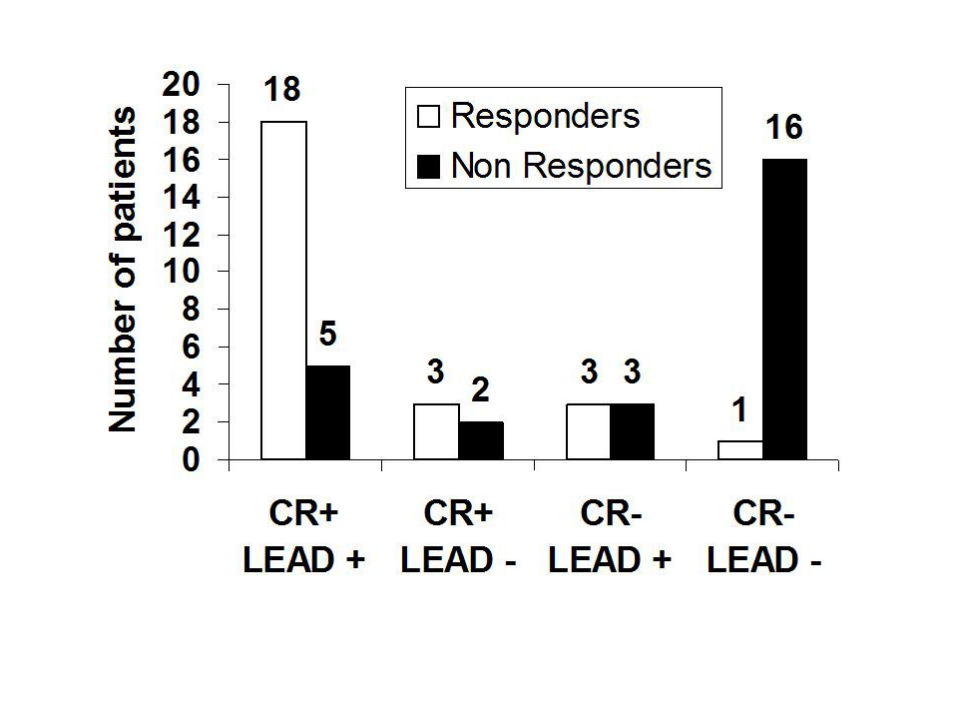
**Number of responders to CRT for 4 different patient categories based on the presence or absence of global contractile reserve (CR+/CR-) in combination with the presence or absence of contractile reserve in the region of the pacing lead (LEAD+/LEAD-)**.

**Figure 4:**
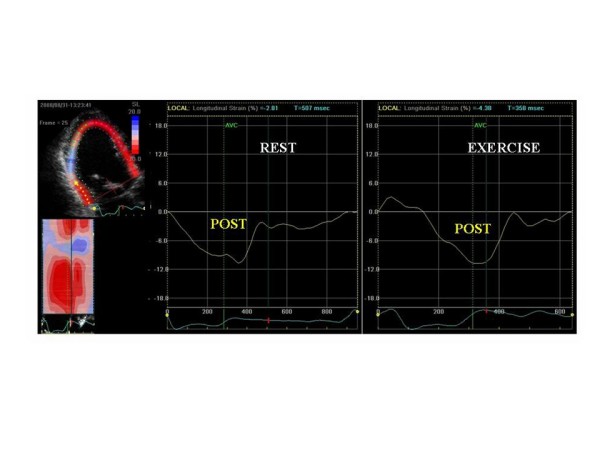
**Example of a patient without contractile reserve in the region of the lead pacing**. There is no increase in 2D speckle tracking strain at exercise in the posterolateral wall (POST).

During exercise, the extent of improvement in regional strain (4.2 ± 1.6% vs 0.44 ± 3.5; p < 0.0001) was greater in patients who presented a significant increase in forward stroke volume. By multivariate logistic regression analysis, the presence of contractile reserve in the LV lead region (OR 0.6: 95% CI 0.39–0.94; p = 0.025) and the global improvement in ejection fraction at exercise (OR 0.59: 95% CI 0.41–0.87; p = 0.008) emerged as independent predictors of response to CRT. An exercise increase in strain values of the target LV lead wall by > 2% (ROC-Curve) yielded a good sensitivity (92%) and specificity (73.1%) for predicting LV reverse remodelling after CRT (AUC 0.89). Among responders, 18 patients had both LV dyssynchrony and regional contractile reserve at exercise (Figure 3). Only 1 out of the 17 patients with no significant local and global contractile reserve responded to CRT. Figure 4 showed a patient without posterolateral contractile reserve during exercise.

## Discussion

The response to CRT is likely modulated by multiple factors. The present study confirms that response to CRT largely depends not only on the extent of LV dyssynchrony and the severity of mitral regurgitation but also on the possibility offers to the LV to recruit function, namely the presence of contractile reserve. The probability to acutely respond to CRT is highly likely in patients with moderate to severe mitral regurgitation, major LV dyssynchrony and significant contractile myocardial recruitment – global and in the LV lead target site – at exercise.

### Role of LV dyssynchrony

Several studies have demonstrated that the major predictor of responsiveness to CRT is mechanical rather than electrical dyssynchrony [6,13,16,17]. Among available techniques, tissue Doppler imaging has emerged as the most practical method for assessing LV dyssynchrony. As reported previously, we found that a significant mechanical delay between the basal segments of the LV, mainly the septum and the lateral wall, on tissue Doppler was highly predictive for response to CRT [6,18]. Patients with a delay ≥ 65 ms showed a significant improvement in forward stroke volume under CRT. However, although this beneficial effect was rarely observed in patients with a delay < 65 ms, 34% (9 of 26) of the patients with severe LV dyssynchrony did not respond to CRT. This emphasizes that other factors play a role; LV dyssynchrony is necessary but not sufficient for CRT response. Indeed, delayed wall motion is mainly a marker of myocardial dysfunction and could be exhibited in viable and scar tissue according to loading conditions [19,20].

### Effect of mitral regurgitation

Response to CRT might be modulated by the presence of functional mitral regurgitation before implantation. Few authors have shown that patients with severe mitral regurgitation have less chance of improving with CRT [3]. However, these studies included a limited number of patients. In the CARE-HF study, a randomized trial including a large number of patients, it was, conversely, shown that patients who were not improved were likely to have no significant mitral regurgitation as compared with responders [7]. The results of the present study confirm and extend this observation. Indeed, we found that a mitral effective regurgitant orifice > 15 mm^2 ^was associated with a higher likelihood of response to CRT. Patients with more severe mitral regurgitation have more LV asynchrony (98 ± 49 ms vs 67 ± 36 ms; p = 0.019). The interplay between LV dyssynchrony and mitral regurgitation is complex [4,21-23]. In fact, the degree of mitral regurgitation is directly correlated to the asynchronous activation of papillary muscles and the decrease in mitral valve closing forces (mainly the consequence of global LV dyssynchrony). It could thus be expected that the beneficial effect of CRT would be greater in patients with significant mitral regurgitation.

### Myocardial reserve and response to CRT

In patients with depressed LV function, the identification of contractile reserve during stress echocardiography has been shown to provide important prognostic information in heart failure patients [24,25]. More specifically, in patients referred to CRT, few authors have reported that the presence of residual myocardial viability might modulate the response to CRT [11,14,26-30]. Contractile reserve is a specific marker of underlying myocardial viability, which can be reliably assessed during semi-supine exercise echocardiography [12]. To the best of our knowledge, this study was the first to examine the value of exercise echocardiography for predicting response to CRT. Significant contractile myocardial recruitment – global and in the LV lead target site – characterized patients who presented the greater rise in forward stroke volume. An increase in LV ejection fraction by ≥ 6.7% and/or an increase in local strain by ≥ 2% were found to be predictive for response to CRT. Moreover, the extent of LV global contractile reserve was related to the percentage of acute changes in forward stroke volume. These data confirm and extend previous studies by demonstrating that a substantial amount of recruitable myocardium is needed to obtain improvement in LV function after CRT. It could be thus argued that in case of advanced myocardial remodeling process, fibrosis and loss of contractile material may severely altered myocardial conduction and contractile properties, which might in turn impede efficient biventricular pacing [31].

### Study limitations

Some limitations should be acknowledged. Our data pertain only to patients with current criteria for CRT implantation. The population studied was not completely homogenous since it was composed of patients with myocardial dysfunction of ischaemic and non ischaemic origin. However, this represents our daily patients referred for CRT. Moreover, biventricular pacing is still a challenging therapy in both settings.

## Conclusion

Heart failure patients referred to CRT have less chance of improving under therapy if they have no significant mitral regurgitation, no LV dyssynchrony and no contractile myocardial recruitment – global and in the LV lead target site – at exercise. It might thus be proposed that the assessment of these parameters would be routinely performed before submitting the patients to CRT.

## Competing interests

The authors declare that they have no competing interests.

## Authors' contributions

All authors participated in the conception and design of the study (MM, MS, BC, PM, EN, LP, PL). The other authors played an important role in the analysis and interpretation of data (MM, MS, BC). The last author wrote the paper (PL). All authors revised the manuscript critically for important intellectual content and gave final approval for submission (MM, MS, BC, PM, EN, LP, PL).

## Supplementary Material

Additional file 1 Case 1: a non-responder with moderate mitral regurgitation, no LV dyssynchrony and transmural necrosis in the posterolateral region. Under CRT, no significant change in mitral regurgitation was observed. Click here for file

Additional file 2 Case 1: a non-responder with moderate mitral regurgitation, no LV dyssynchrony and transmural necrosis in the posterolateral region. Under CRT, no significant change in mitral regurgitation was observed. Click here for file

Additional file 3 Case 2: a responder with severe mitral regurgitation, LV dyssynchrony and viability in the posterolateral region. Under CRT, mitral regurgitation decreased and the aortic TVI increased. Click here for file

Additional file 4 Case 2: a responder with severe mitral regurgitation, LV dyssynchrony and viability in the posterolateral region. Under CRT, mitral regurgitation decreased and the aortic TVI increased. Click here for file

Additional file 5 Cases 1 and 2 responders. Click here for file
