# Supplementary material for: Impact of contractile reserve on acute response to cardiac resynchronization therapy
Source: Cardiovasc Ultrasound. 2008 Dec 31;6:65. doi: 10.1186/1476-7120-6-65 (PMC2615753; doi:10.1186/1476-7120-6-65)
Supplement: Additional file 5 — Cases 1 and 2 responders. [file 1476-7120-6-65-S5.ppt]

## Slide 1
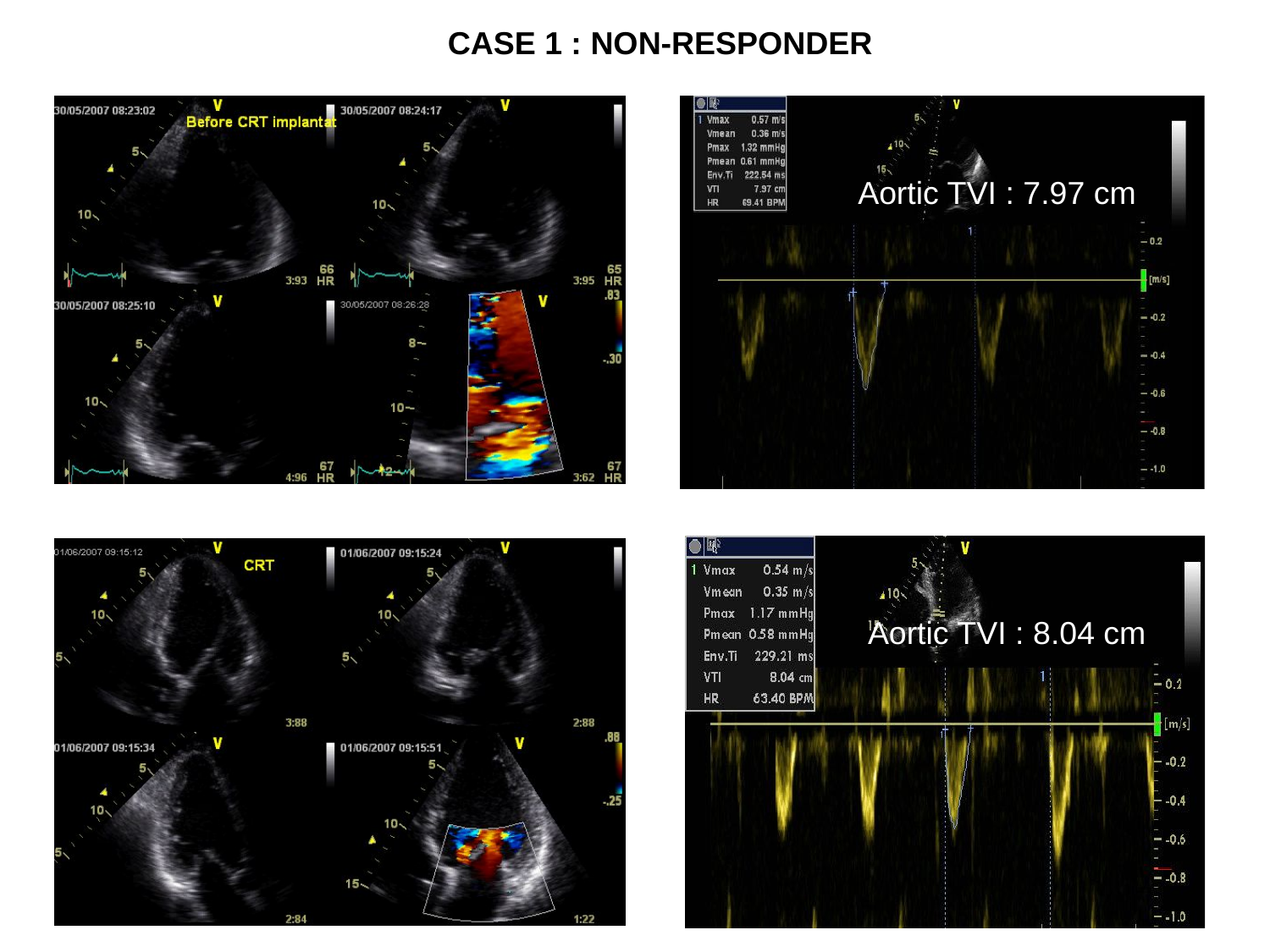

CASE 1 : NON-RESPONDER
Aortic TVI : 7.97 cm
Aortic TVI : 8.04 cm

## Slide 2
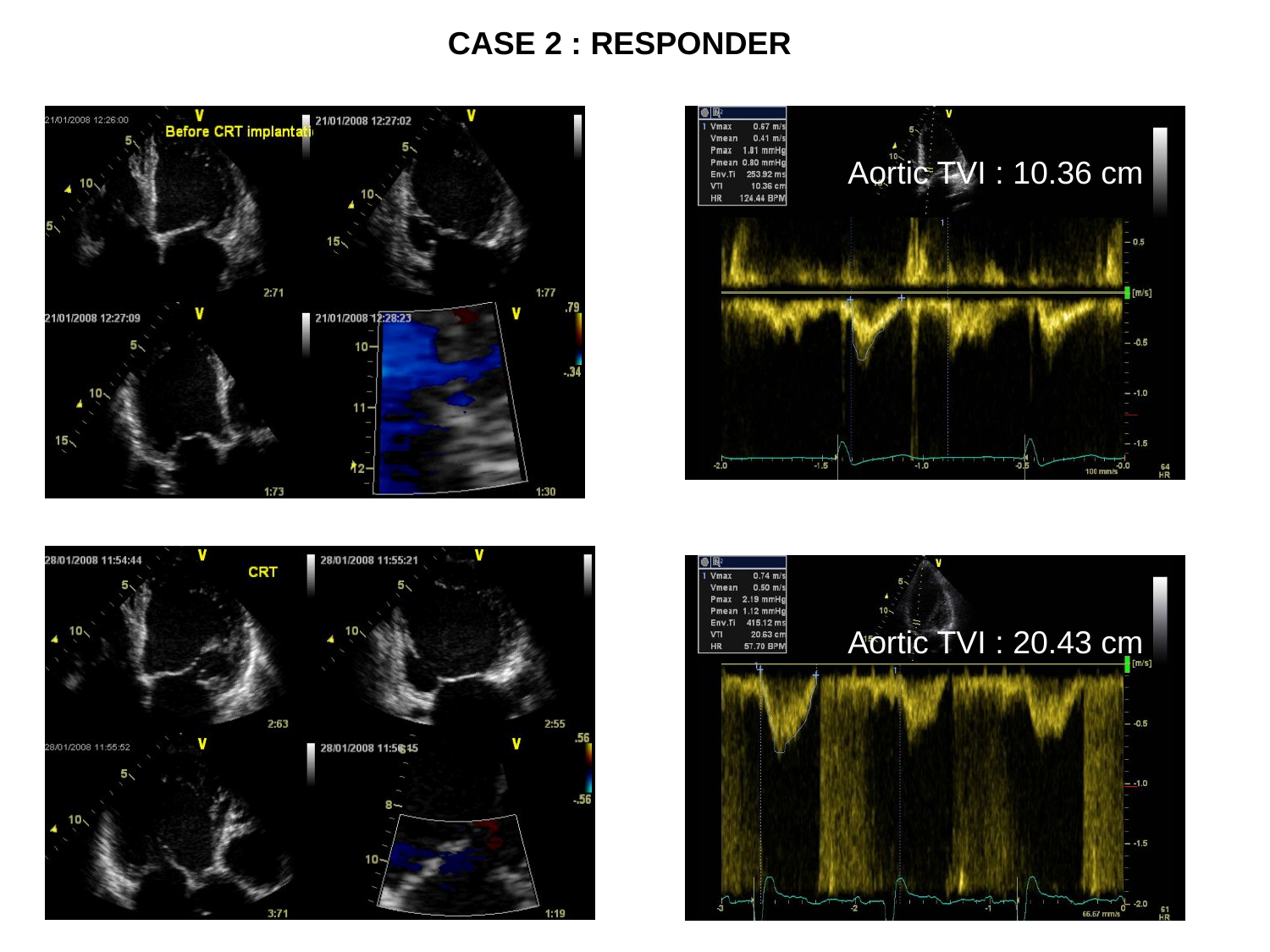

CASE 2 : RESPONDER
Aortic TVI : 10.36 cm
Aortic TVI : 20.43 cm
